# Bone mineral density and body composition in postmenopausal women with psoriasis and psoriatic arthritis

**DOI:** 10.1186/ar3240

**Published:** 2011-02-07

**Authors:** Paulo G Pedreira, Marcelo M Pinheiro, Vera L Szejnfeld

**Affiliations:** 1 Rheumatology Division, Federal University of São Paulo, UNIFESP/Paulista School of Medicine, EPM. 740, Botucatu Street, 04023-900, São Paulo-SP, Brazil

## Abstract

**Introduction:**

The aim of the present study was to compare bone mineral density (BMD) and body composition (BC) measurements as well as identify risk factors for low BMD and osteoporotic fractures in postmenopausal women with psoriasis (Ps) and psoriatic arthritis (PsA).

**Methods:**

A cross-sectional study was carried out in 45 PsA women, 52 Ps women and 98 healthy female controls (HC). Clinical risk factors for low bone density and osteoporotic fracture were evaluated by a specific questionnaire. An X-ray absorptiometry (DXA) at the lumbar spine, total femur and total body was performed on all patients. Skin and joint outcomes were measured by specific tools (PASI, HAQ and DAS28). Morphometric vertebral fractures were evaluated by lumbar and thoracic spine X-ray, according to Genant's method.

**Results:**

There were no significant differences in age, body mass index (BMI), total lean mass and bone mineral density among the groups. However, the PsA group had a significantly higher body fat percentage (BF%) than the Ps and HC groups. Osteoporotic fractures were more frequently observed in PsA and Ps groups than in the HC group (*P *= 0.01). Recurrent falls and a longer duration of disease increased the risk of fracture (odds ratio (OR) = 18.3 and 1.08, respectively) in the PsA group (*P *= 0.02). Disability was the main factor related to osteoporotic fracture in the Ps group (odds ratio (OR) = 11.1) (*P *= 0.02).

**Conclusions:**

Ps and PsA patients did not present lower BMD. However, they had a higher prevalence of osteoporotic fractures and higher risk of metabolic syndrome. Patients with a longer duration of disease, disability and recurrent falls need preventive measures.

## Introduction

Psoriasis (Ps) and psoriatic arthritis (PsA) are chronic, immuno-mediated inflammatory diseases characterized by abnormal expressions of keratinocytes, with actions of interferon (IFN)-γ, tumor necrosis factor (TNF)-α, TNF-β, transforming growth factor (TGF)-β, interleukin (IL)-1, IL-6, IL-8 and IL-17 [1-3] or activation of Th2 inflammatory response, releasing TNF-α, IL-1 and IL-6, as well as proliferation and neovascularization of the synovial [4,5].

According to Colucci *et al*., there is greater *in vitro *expression of TNF-α and receptor activator of nuclear factor-kappa ligand (RANKL) in T and B cells of the peripheral blood as well as T cells and fibroblasts in the synovial fluid of patients with PsA [6]. Hofbauer *et al*. found an increase in serum RANKL in these patients and significant reduction of the osteoclastogenesis after treatment with TNF inhibitors [7]. Ng *et al*., evaluating rheumatoid arthritis (RA) and PsA patients before and after one year of anti-TNF therapy, demonstrated increased bone density and bone formation markers in both groups as well as reduction of bone resorption markers [8]. Nymann *et al*. found a decrease of spine BMD in patients with palmoplantar pustulosis [9]. However, this same correlation was not observed in Ps [10] or PsA patients [11,12].

Higher release of IL-1, IL-2, IL-6, IFN-γ and TNF-α is associated with lean mass loss in patients with AIDS, cancer, chronic obstructive pulmonary disease, kidney failure, RA, acute myocardial infarction [13,14]. Inflammatory cytokines activate the muscle proteolytic system, stimulate the release of cortisol and catecholamines, induce lipolysis and β-oxidation, as well as higher synthesis of very low density lipoprotein (VLDL) and triglycerides and an increase of fat mass [15]. The growth hormone (GH) and type I insulin-like growth factor (IGF-I) increase the protein synthesis by myocites. Toussirot *et al*. found higher serum leptin in RA patients as well as a positive correlation with fat mass and negative association with lean mass. However, the GH concentration was increased and IGF-I did not differ from the control group [16]. Westhovens *et al*. found a significant reduction of total body BMD and lean mass as well as an increase of fat mass in RA patients when compared to HC [17]. Marcora *et al*. demonstrated a significant decrease of lean mass and muscle strength in men with ankylosing spondylitis (AS) [18]. Briot *et al*. verified higher spine and femur BMD and fat mass after treatment with etanercept and infliximab in AS patients [19]. Saraceno and Gisondi demonstrated an increase of body weight in Ps and PsA patients treated with etanercept, adalimumab and infliximab [20,21].

The aim of the present study was to analyze bone density and body composition in postmenopausal women with psoriasis, psoriatic arthritis and in healthy controls in order to identify risk factors for low bone mass, fractures and changes of body composition.

## Materials and methods

A cross-sectional study was carried out in 45 PsA women, 52 Ps women and 98 HC. The diagnosis of PsA was defined by the Classification Criteria for Psoriatic Arthritis (CASPAR) [22]. PsA patients were in treatment at the Rheumatology Division of Federal University of São Paulo (UNIFESP)/Paulista School of Medicine (EPM) and Sao Paulo Public Servants' Hospital. Ps patients were regularly followed-up at the UNIFESP/EPM Dermatology Outpatient Clinic. The control group was paired for gender, age, BMI, ethnic background and socioeconomic class. The study was approved by the Committee of Medical Ethics in Research of UNIFESP/EPM. All patients agreed to participate in the study and signed the informed consent form. Clinical risk factors to low bone density and osteoporotic fracture were evaluated by a specific questionnaire based on the European Vertebral Osteoporosis Study (EVOS) that included details about anthropometric data, personal and medical history, gynecological information, concomitant medications, diet, smoking and physical activity [23].

Bone mineral density measurements were performed at lumbar spine and proximal femur, using a dual-energy X-ray densitometer (GE-Lunar Radiation Corporation, DPX MD + model, Madison, WI, USA). The standard technical procedure was followed according to the manufacturer's instructions. The US National Health and Nutrition Examination Survey (NHANES III) database was adopted. The right femur was studied in all individuals, except in those with a hip fracture, prosthesis or severe arthritis on this side, in which cases the left femur was evaluated. The coefficient of variation at our service is 3% for the femur and 2% for the lumbar spine. Total body densitometry was performed to evaluate body composition (lean, fat and bone mass). The skeletal muscle mass index (SMMI) was calculated as the ratio of the sum of the lean mass of the arms and legs (kg) divided by the height squared (m^2^). The same calculation was performed for the skeletal fat mass index (kg/m^2^), considering the fat mass of the arms and legs. Sarcopenia was defined by a SMMI below 5.45 [24]. The coefficient of variation for the total body is 3%. In order to differentiate the terms from BMI, the procedure described by Giles *et al*. was adopted, in which a BMI below 18.50 kg/m^2 ^is considered underweight; a BMI between 18.50 and 24.99 kg/m^2 ^is the ideal weight range; a BMI between 25 and 29.99 kg/m^2 ^is considered overweight; and a BMI equal to or greater than 30 kg/m^2 ^is obese [25]. Overfat was defined if total body fat exceeding 41%, measured by DXA, in White women under 60 years old or 43% for women with 60 years or older [25,26]. Obese sarcopenia was defined as a patient fulfilling overfat and sarcopenia criteria [25].

The Psoriasis Area Severity Index (PASI) was used to assess the extent and severity of skin involvement in patients with Ps and PsA [27]. The Health Assessment Questionnaire (HAQ) was applied to determine the degree of temporary or definitive functional impairment [28]. Disease Activity Score 28 (DAS28) and Psoriatic Arthritis Response Criteria (PsARC) were used to measure joint activity in PsA patients [29,30].

A thoracic and lumbar spine X-ray was performed to identify and classify vertebral deformities or fractures, accordingly Genant's criteria [31].

Secondary osteoporosis, osteoarthritis that could compromise the bone mineral density measurements or previous diagnosis of sarcopenia were considered exclusion criteria as well as patients with Steinbroker functional class IV; use of insulin, statins, GH, anabolic agents, hormonal therapy, TNF blockers or vitamin D; cognitive impairment; cancer or active HIV infection.

### Statistical analysis

The descriptive summary is presented in the form of mean ± standard deviation. Levene's test was used to test the homogeneity of the sampling. The multiple comparisons among the groups were done with analysis of variance (ANOVA), Tukey's *post hoc *test, Kruskal-Wallis test or Mann-Whitney test. Chi-square test or Fisher's exact test were used for the qualitative variables. BMD and BC measurements were considered as dependent variables. Pearson's correlation coefficient was calculated between quantitative variables that satisfied the conditions of normality, as determined by the Kolmogorov-Smirnov test. Otherwise, Spearman's correlation coefficient was preferred. Logistic regression models were used for binary categorical variables (low-impact fracture). In the multivariate regression, two models were performed for bone density separately (spine and femur BMD). Statistical adjustments were done for age and weight in all tests. The level of significance was set as *P *< 0.05. The analyses were performed using the Statistical Package for Social Sciences (version 15) (IBM, Chicago, Illinois, USA).

## Results

A total of 195 postmenopausal women were studied (45 PsA patients, 52 Ps patients and 98 HC), paired for age and BMI (Table 1).

**Table 1:** Age, anthropometric data and clinical characteristics of the women studied

	PsA *N = *45	Ps *N = *52	Control *N = *98	*P*
Age (years)	60.5 ± 8.7	61.4 ± 9.1	60.1 ± 8.4	0.95
Weight (kg)	69.4 ± 8.9	71.1 ± 14.2	67.8 ± 11.2	0.19
Height (m)	1.50 ± 0.7	1.50 ± 0.6	1.55 ± 0.6	0.97
BMI (kg/m^2^)	29.1 ± 3.6	26.6 ± 6.2	28.1 ± 4.54	0.19
Within ideal weight range *				
N (%)	7 (15.6)	9 (17.3)	25 (25.5)	0.29
Overweight^†^				
N (%)	17 (37.8)	22 (42.3)	40 (40.8)	0.9
Obese^‡^				
N (%)	21 (46.7)	21 (40.4)	33 (33.7)	0.31
Ps Duration (years)	24.8 ± 16.2	21.8 ± 17.8	-	0.39
PsA Duration (years)	11 ± 10.5	-	-	-
PASI	2.2 ± 3.3	3.2 ± 3.4	-	0.22
HAQ	0.7 ± 0.5^§||^	0.2 ± 0.3	0.1 ± 0.2	<0.001
DAS28	3.7 ± 1.4	-	-	-
PsARC				
N° of swollen joints	3.2 ± 2.9	-	-	-
N° of painful joints	3.5 ± 3.4	-	-	-
Global evaluation of patient (VAS)	4 ± 2.8	-	-	-
Global evaluation of physician (VAS)	3.3 ± 2.4	-	-	-
Years since of menopause	12.7 ± 9.9	15.4 ± 10.9	12.1 ± 8.6	0.22

*BMI 18.50 - 24.99 kg/m^2^; ^†^BMI 25 - 29.99 kg/m^2^; ^‡^BMI ≥ 30 kg/m^2^

DAS28, disease activity score 28; HAQ, health assessment questionnaire; PASI, psoriasis area severity index; PsA, psoriatic arthritis; Ps, psoriasis; PsARC, psoriatic arthritis response criteria; VAS, visual analogue scale.

^§^*P *< 0.001 (PsA vs. Ps)

^||^*P *< 0.001 (PsA vs. control)

There were no significant differences in the parameters related to clinical characteristics of the disease, such as duration of skin condition and PASI. However, time of disease was two-fold greater in Ps patients. Ungual involvement was more frequent in the PsA group (64.4%) than the Ps group (36.5%) (*P *= 0.006). PsA patients had more disability than the Ps group and healthy controls (*P *< 0.001). On average, joint activity was more severe than skin activity. Besides, there was no significant difference in years since menopause (Table 1). No patient had undergone hormonal replacement therapy for longer than three months.

Hypertension was highly prevalent, especially among PsA and Ps patients. The Ps group had more cases of type 2 diabetes mellitus and dyslipidemia, without statistically significant differences between groups.

PsA patients had longer duration of glicocorticosteroids use than Ps women (9.6 ± 22.8 and 4.8 ± 13.2 months, respectively; *P *= 0.14). There was no statistically significant difference in family history of osteoporosis or hip fracture between PsA and Ps groups as well as recurrent falls in the last year. However, falls were more frequent in the patients than in the control group (*P *< 0.001). PsA patients had more fractures (33.3%) than the Ps group (28.8%) (*P *= 0.018). Fragility fractures were more prevalent among the Ps and PsA groups than the HC (*P *= 0.001). The PsA group had more morphometric vertebral fractures than the Ps and control groups (*P *= 0.06). Dairy product intake was low, but similar, between the three groups. Alcoholic beverage consumption was infrequent with no significant difference between groups. No patient reported the use of illicit drugs. A current smoking habit was more frequent in Ps patients (21.2%) than in PsA women (11.1%) (*P *= 0.11).

Total body, spine and femur BMC and BMD measurements were not different among the three (Table 2). On the other hand, the PsA group presented almost significantly less total lean mass (TLM) than the Ps and HC group (*P *= 0.06). All groups had a large amount of total body fat, especially patients with PsA (*P *= 0.04). Total body fat (TBF) above expected values for individuals of the same gender and age occurred in the majority of PsA (84.4%) and Ps (65.4%) patients (*P *= 0.02). Overfat was significantly more identified in the PsA group than in the other two groups (*P *= 0.04), mainly with android distribution. Moreover, all patients with PsA and sarcopenia were obese (Table 3).

**Table 2:** Bone density of total body, lumbar spine and proximal femur among the women studied

	PsA	Ps	Control	*P*
	*N = *45	*N = *52	*N = *98	
Total Body				
BMC (g)	2140 ± 282	2161 ± 287	2207 ± 304	0.40
BMD (g/cm^2^)	1.118 ± 0.09	1.118 ± 0.1	1.117 ± 0.08	0.99
Lumbar Spine (g/cm^2^)	1.058 ± 0.16	1.054 ± 0.13	1.058 ± 0.15	0.98
Total Femur (g/cm^2^)	0.934 ± 0.13	0.922 ± 0.13	0.949 ± 0.12	0.46

BMC, bone mineral content; BMD, bone mineral density; Ps, psoriasis; PsA, psoriatic arthritis.

**Table 3:** Body composition, overfat and sarcopenia according to the body fat normality curves measured by DXA

	PsA	Ps	Control	*P*
	*N = *45	*N = *52	*N = *98	
TLM (kg)	35.28 ± 3.96	37.45 ± 5.32	35.93 ± 4.81	0.06
TBF (%)	46 ± 5.7*	43.8 ± 6.2	43.4 ± 5.5	0.04
Within normality (%)	15.6^†‡^	34.6	37.8	0.02
Above normality (%)	84.4^†‡^	65.4	62.2	0.02
Overfat (%)	86.7 ^|| §^	61.5	58.2	0.03
Sarcopenia (%)	11.1	5.8	6.1	0.50
Obesity-sarcopenia (%)	11.1	3.8	4.1	0.19

DXA, dual X-ray absorptiometry; Ps, psoriasis; PsA,: psoriatic arthritis; TBF, total body fat; TLM, total lean mass.

*P = 0.03 (PsA *vs*. control)

^†^*P *< 0.05(PsA vs Ps)

^‡^*P *< 0.05 (PsA vs control)

^§ ^*P *< 0.01 (PsA *vs*. Ps)

^|| ^*P *< 0.01 (PsA *vs*. control)

In the PsA group, spine BMD was negatively correlated with years since menopause. TLM and TBF were positively correlated with weight and BMI (Table 4). In the Ps group, spine BMD had positive correlation with weight and TLM. Femur BMD presented a positive correlation with weight, BMI and TLM and inverse correlation with age. TLM and TBF were positively correlated with weight and BMI (Table 5).

**Table 4:** Correlations between anthropometric data, clinical findings and bone density measurements in patients with psoriatic arthritis

	Vertebral	Femur	TLM	TBF
	BMD	BMD		
Age r*	-0.05	-0.09	0.11	0.02
(*P*)	(0.76)	(0.53)	(0.45)	(0.86)
Weight r*	0.27	0.13	**0.63**	**0.59**
(*P*)	(0.78)	(0.39)	**(<0.001)**	**(<0.001)**
BMI r*	0.16	0.20	**0.35**	**0.63**
(*P*)	(0.30)	(0.17)	**(0.02)**	**(<0.001)**
Duration of Ps r*	0.23	0.02	0.13	-0.10
(*P*)	(0.13)	(0.88)	(0.37)	(0.52)
Duration of PsA r^†^	0.14	0.09	0.14	0.15
(*P*)	(0.34)	(0.55)	(0.35)	(0.34)
Duration of menopause r^†^	**-0.29**	-0.14	0.07	-0.04
(*P*)	**(0.04)**	(0.37)	(0.64)	(0.79)
HAQ r^†^	0.04	0.01	-0.27	-0.02
(*P*)	(0.8)	(0.93)	(0.08)	(0.91)
PASI r^†^	0.08	-0.13	0.13	-0.15
(*P*)	(0.6)	(0.39)	(0.38)	(0.31)
DAS 28 r*	-0.13	-0.19	-0.12	0.12
(*P*)	(0.39)	(0.2)	(0.41)	(0.43)
PsARC for swollen joints r^†^	0.13	-0.16	-0.01	0.02
(*P*)	(0.41)	(0.29)	(0.94)	(0.88)
PsARC for painful joints r^†^	-0.13	-0.03	0.07	0.01
(*P*)	(0.38)	(0.86)	(0.63)	(0.93)
PsARC (VAS) - patient r^†^	0.05	-0.01	-0.15	0.08
(*P*)	(0.74)	(0.91)	(0.32)	(0.57)
PsARC (VAS) - physician r^†^	0.01	0.01	-0.62	0.05
(*P*)	(0.93)	(0.94)	(0.69)	(0.75)
TLM r*	0.19	0.15	1	-0.22
(*P*)	(0.2)	(0.33)		(0.15)
TBF r*	0.11	-0.006	-0.22	1
(*P*)	(0.48)	(0.97)	(0.15)	

r*, Pearson's correlation; r^†^, Spearman's correlation

**Table 5:** Correlations between anthropometric data, clinical findings and bone density measurements in patients with psoriasis

	Vertebral	Femur	TLM	TBF
	BMD	BMD		
Age r*	-0.21	**-0.32**	-0.07	0.15
(*P*)	(0.13)	**(0.02)**	(0.64)	(0.3)
Weight r^†^	**0.29**	**0.45**	**0.72**	**0.76**
(*P*)	**(0.04)**	**(<0.001)**	**(<0.001)**	**(<0.001)**
BMI r^†^	0.16	**0.43**	**0.55**	**0.74**
(*P*)	(0.26)	**(<0.001)**	**(<0.001)**	**(<0.001)**
Duration of Ps r^†^	-0.22	0.07	0.00	-0.09
(*P*)	(0.12)	(0.96)	(0.97)	(0.53)
Duration of menopause r^†^	-0.08	-0.2f	-0.18	-0.06
(*P*)	(0.55)	(0.09)	(0.19)	(0.96)
HAQ r^†^	-0.08	0.09	0.1	0.21
(*P*)	(0.55)	(0.48)	(0.48)	(0.14)
PASI r^†^	0.06	0.05	0.05	-0.87
(*P*)	(0.68)	(0.72)	(0.73)	(0.54)
TLM r^†^	**0.28**	**0.56**	1	0.19
(*P*)	**(0.04)**	**(<0.001)**		(0.18)
TBF r^†^	0.06	0.14	0.19	1
(*P*)	(0.65)	(0.33)	(0.18)	

r*, Pearson's correlation; r^†^, Spearman's correlation

In the PsA group, the final logistic regression model revealed an 8% increase in the risk of fragility fracture for each year of joint disease. Furthermore, the number of falls increased from 2- to 18-fold the risk of fracture in patients with recurring events. In the Ps group, there was an 11-fold increase in the risk for each lower unit of functional capacity (Table 6).

**Table 6:** Final logistic regression model for low-impact fraction among patients with psoriatic arthritis and psoriasis

	Group	OR	95% CI	*p*
Duration of PsA	PsA	1.08	1.01 to 1.16	0.01
N° of falls	PsA			
1 to 3		2.5	0.5 to 12.4	0.02
				
>3		18.3	1.5 to 217.7	0.02
HAQ	Ps	11.1	15.5 to 84.2	0.02

HAQ, health assessment questionnaire; Ps, psoriasis; PsA, psoriatic arthritis.

The final multivariate regression model showed a reduction of 0.01 g/cm^2 ^in spine BMD for each year since menopause to PsA patients. However, none of the variables studied were significantly associated with femur BMD. In the Ps group, there was an increase of spine and femur BMD for each one-gram increase in the TLM. Moreover, femur BMD was reduced by 0.005 g/cm^2 ^for every year of increasing age.

The total hip or total femur BMD showed better performance than the femoral neck in the final model of multivariate regression in both groups (Ps and PsA). Therefore, it was chosen as the most significant.

## Discussion

Our results did not demonstrate any significant difference in spine or femur BMD in PsA or Ps patients and healthy controls. Lean mass was similar in PsA, Ps and HC groups, although PsA patients had a trend to a lower lean mass than Ps patients. PsA patients had a greater percentage of fat mass than Ps women and HC. These results are similar to those described by Borman *et al*. [11], Dheda *et al*. [32], Millard *et al*. [10] and Grisar *et al*. [33]. However, these authors studied small, non-homogeneous (men, pre- and postmenopausal women) populations and had no adequate control group.

There was also no significant difference among the three groups when applying the 1994 World Health Organization (WHO) criteria for osteoporosis [34]. On the other hand, Frediani *et al*. found a reduction of BMD in two-thirds of PsA patients when compared to HC [35]. Potentially, PsA and Ps patients have higher bone resorption markers and bone loss related to inflammatory process and disability [33]. Similarly, Heim *et al*. observed higher remodeling rates, without trabecular bone loss, in PsA and Ps patients [36]. The authors believe that the same hyperproliferation that occurs in the skin may also occur in bone tissue, thereby causing latent bone disease. Besides, they suggest that Ps patients have partial resistance to the action of vitamin D on dermic fibroblasts. If the same situation occurs in bone tissue, this could explain the accelerated bone remodeling. In the present study, no assessment of bone remodeling markers or of bone biopsies was done.

Other aspects may have also played a protective role regarding the bone density values found in the present study, such as an overweight condition as well as decreased smoking and lower alcohol intake. Dairy product intake was low or inadequate in all groups, and similar to that reported by Pinheiro *et al*. in the Brazilian population [37]. This point demonstrates that calcium intake did not play a relevant role in bone mass of these subjects. Furthermore, few patients were taking glucocorticosteroids or had used them for short periods of time and in low dosages.

Although joint activity among PsA patients has been considered moderate to severe, the skin condition was considered mild in both groups. Moreover, dermatological recommendations for psoriasis, such as exposure to sunlight and ultra-violet light baths, play a beneficial role on bone metabolism due to vitamin D synthesis [38].

Among all the risk factors investigated, only age and years since menopause were determinant of the BMD. Age for femur BMD in Ps patients and years since menopause for spine BMD in PsA postmenopausal women are similar to those reported by Frediani *et al*. [35].

Studies on body composition in inflammatory disease are more frequent in RA or AS than is PsA patients. In general, these studies have shown greater lean mass loss, especially in those with higher activity of disease and disability. The present study demonstrates that the PsA group had a tendency to lower lean mass than Ps women or HC.

There was a positive correlation between lean mass and femur and spine BMD in Ps patients. In the final multivariate regression model, TLM was the most important aspect related to femur BMD, suggesting that exercise and higher strength musculature could be relevant strategies for increase bone mass in these patients. This correlation was not found in PsA patients.

We observed a high percentage of total body fat in all three groups, especially in the PsA group (*P *= 0.04). Giles *et al*. pointed out that RA patients with an overfat phenotype had a greater degree of joint deformity and disability, as well as higher C-reactive protein (CRP) levels. Also, these patients are more sarcopenic and sarcopenic-obese [25]. Although it was not significant, in the present study, there was a higher percentage of sarcopenia in PsA and Ps groups. Surprisingly, all PsA patients with sarcopenia were obese. Both Ps and PsA group had a higher frequency of android fat distribution, which is traditionally associated with a greater risk of cardiovascular disease, metabolic syndrome and diabetes mellitus. Our findings are similar to those described in rheumatoid patients [17]. A psoriasic chronic inflammatory process causes an increase of type II insulin-like growth factor (IGF-II), which is responsible for epidermal cell proliferation and is also implicated in atherogenesis and adipogenesis, as well as an increase in vascular endothelial growth factor (VEGF), which may be associated with angiogenesis and hyperinsulinemic states [39].

Genes associated with Ps and PsA may also be present in patients with metabolic disorders. The psoriasis susceptibility (PSORS) *loci *PSORS2, PSORS3 and PSORS4 are associated with susceptibility *loci *to metabolic syndrome, type 2 diabetes mellitus, familial hyperlipidemia and cardiovascular disease. Individual genes associated with Ps, such as Cyclin-dependent kinase 5 regulatory subunit associated protein 1-like 1 (CDKAL1), are also associated with type 2 diabetes. Likewise, genes involved with a higher risk of cardiovascular disease, such as the isoform ApoE4 of ApoE (Apolipoprotein E), are more prevalent in Ps patients [40]. In the present study, type 2 diabetes mellitus was more frequent in Ps patients. This is similar to the findings described by Sommer *et al.*, who also found greater risk of hypertension and dyslipidemia in these patients [41]. Furthermore, there was a greater prevalence of hypertension in PsA and Ps groups than HC; whereas, dyslipidemia has been more frequent in Ps patients.

Our data suggest that both PsA and Ps patients have a higher risk for developing metabolic syndrome and cardiovascular disease. It is well established that Ps patients have hyperuricemia, which could act as an aggravating factor for metabolic syndrome. Thus, we may suppose greater prevalence of metabolic disease in PsA and Ps patients than in RA or AS patients.

Vertebral and non-vertebral fractures were significantly more prevalent in PsA and Ps patients than in the HC and Brazilian population over 40 years old [42]. Sinigaglia *et al*. found an association among fracture and advanced age, disability, more prolonged disease and more severe disease activity in RA and AS patients [43]. Yes, the sentence is correct. In the present study, longer disease duration and recurrent falls were predictors of low-impact fractures in PsA patients, while disability was predictive of fragility fracture in Ps patients.

As bone mass measurements did not reflect the bone status in Ps and PsA patients, the evaluation of fractures is important. Bone neoformation may be another explanation for the absence of densitometric osteoporosis in PsA patients. However, none of our subjects have had clinical or radiographic axial involvement.

## Conclusions

PsA and Ps patients have higher risk of fragility fracture and it seems that they also have a higher risk of developing metabolic disease, especially those patients with longer disease duration, disability and recurrent falls.

## Abbreviations

AIDS: acquired immune deficiency syndrome; ANOVA: analysis of variance; ApoE: apolipoprotein E; AS: ankylosing spondylitis; BC: body composition; BF: body fat; BMD: bone mineral density; CASPAR: Classification Criteria for Psoriatic Arthritis; CDKAL1: cyclin-dependent kinase 5 regulatory subunit associated protein 1-like 1; CRP: C-reactive protein; DAS28: Disease Activity Score 28; DXA: dual x-ray absorptiometry; EPM: Paulista School of Medicine; EVOS: European Vertebral Osteoporosis Study; GH: growth hormone; HC: healthy controls; HAQ: Health Assessment Questionnaire; HIV: human immunodeficiency virus; IFN: interferon; IGF: insulin-like growth factor; IL: interleukin; NHANES III: National Health and Nutrition Examination Survey; OR: *Odios ratio*; Ps: psoriasis; PsA: psoriatic arthritis; PsARC: Psoriatic Arthritis Response Criteria; PASI: Psoriasis Area Severity Index; PSORS: psoriasis susceptibility locus; RA: rheumatoid arthritis; RANKL: receptor activator of nuclear factor-kappa ligand; SMMI: skeletal muscle mass index; TBF: total body fat; TGFβ: transforming growth factor beta; Th: T-helper; TLM: total lean mass; TNF: tumor necrosis factor; UNIFESP: Federal University of São Paulo; VAS: visual analogue scale; VEGF: vascular endothelial growth factor; VLDL: very low density lipoprotein; WHO: World Health Organization.

## Competing interests

The authors declare that they have no competing interests.

## Authors' contributions

PGG conducted data collection, interpretation and analysis of the data and drafted the manuscript. MMP participated in study design, analyzed bone scan DXA as well as spine X-ray for radiographic fractures, interpreted and analyzed the data and helped to draft the manuscript. VLS participated in study design, interpretation and analysis of the data and helped to draft the manuscript. All authors critically reviewed, contributed to and approved the final manuscript.
